# Analysis and modelling of motility of cell populations with MotoCell

**DOI:** 10.1186/1471-2105-10-S12-S12

**Published:** 2009-10-15

**Authors:** Concita Cantarella, Leandra Sepe, Francesca Fioretti, Maria Carla Ferrari, Giovanni Paolella

**Affiliations:** 1 DBBM Dipartimento di Biochimica e Biotecnologie Mediche, Universita' di Napoli FEDERICO II, Via S. Pansini 5, 80131 Napoli, Italy; 2 S.E.M.M. – European School of Molecular Medicine – Naples site, Italy; 3 CEINGE Biotecnologie Avanzate scarl, Via Comunale Margherita 482, 80145 Napoli, Italy

## Abstract

**Background:**

Cell motility plays a central role in development, wound-healing and tumour invasion. Cultures of eucariotic cells are a complex system where most cells move according to 'random' patterns, but may also be induced to a more coordinate migration by means of specific stimuli, such as the presence of chemical attractants or the introduction of a mechanical stimulus. Various tools have been developed that work by keeping track of the paths followed by specific objects and by performing statistical analysis on the recorded path data. The available tools include desktop applications or macros running within a commercial package, which address specific aspects of the process.

**Results:**

An online application, MotoCell, was developed to evaluate the motility of cell populations maintained in various experimental conditions. Statistical analysis of cell behaviour consists of the evaluation of descriptive parameters such as average speed and angle, directional persistence, path vector length, calculated for the whole population as well as for each cell and for each step of the migration; in this way the behaviour of a whole cell population may be assessed as a whole or as a sum of individual entities. The directional movement of objects may be studied by eliminating the modulo effect in circular statistics analysis, able to evaluate linear dispersion coefficient (R) and angular dispersion (S) values together with average angles. A case study is provided where the system is used to characterize motility of RasV12 transformed NIH3T3 fibroblasts.

**Conclusion:**

Here we describe a comprehensive tool which takes care of all steps in cell motility analysis, including interactive cell tracking, path editing and statistical analysis of cell movement, all within a freely available online service. Although based on a standard web interface, the program is very fast and interactive and is immediately available to a large number of users, while exploiting the web approach in a very effective way. The ability to evaluate the behaviour of single cells allows to draw the attention on specific correlations, such as linearity of movement and deviation from the expected direction. In addition to population statistics, the analysis of single cells allows to group the cells into subpopulations, or even to evaluate the behaviour of each cell with respect to a variable reference, such as the direction of a wound or the position of the closest cell.

## Background

Cell migration is involved, at various extents, in fundamental processes as embryo development and organogenesis, organism growth and survival and response to pathological situations. In the developing embryo, coordinated cell migration involves movement of cells of different origin throughout the embryo, over short and long distance paths; defects of migration at all stages of development lead to severe embryonic malformations and result in drastic overall consequences [1]. In adult organisms, cell movement is essential in wound-healing, where epidermal repair, initiated by the progressive extension of a tongue of epidermal cells, results in complete closure of the wound. Cell migration is also involved in inflammation and atherosclerosis and is responsible for primary invasion of cancer cells and metastatization [2].

Cell cultures are often studied as model systems for movement, as a population of cells growing *in vitro *moves on the culture surface using the same complex membrane machinery used by cells in vivo. A large amount of experimental work, carried out in many laboratories, has provided a good understanding of the processes and interactions which control cell motility. Integrin receptors, focal adhesion structures, cytoskeletal elements and signalling molecules are important players both *in vivo *and *in vitro *[3-5]. Mathematical and computational methods have also been developed to model specific aspects of movement, such as formation of membrane protrusions and actin dynamics, [6,7]. In addition, cell movement has been studied with approaches which take into account the whole cell, where mechanical events such as protrusion, contraction and relaxation all contribute to produce cell displacement [8-10].

The behaviour of the cell population has also been analyzed. In absence of particular conditions, cells move on the culture plate over smaller or larger distances, depending on the cell type and culture conditions, and in all possible directions; the culture can also be exposed to specific stimuli which can affect both speed and direction. Video time-lapse microscopy is used to dynamically study the phenomenon. By acquiring multiple images of the same field over time, a stack of images is produced which together describe migration in two, or even three, dimensions. Cell motion is evaluated by tracking subsequent cell positions either manually, by marking, with the assistance of a computer, the positions assumed by individual cells in stacks of recorded images, or automatically. Cell tracking algorithms may not be as accurate as manual recording, but require less time and may be used for the analysis of a large number of cells. They use simple methods which calculate the position assumed by a labelled cell or the nucleus, after segmenting the image on the basis of intensity [11], or with more sophisticated methods, where subsequent deformations of an initial contour model are used to identify cell boundaries in the next frames [12]. Paths are typically described by list of coordinates corresponding to the trail followed by moving cells, and are subsequently analyzed in order to extract descriptive parameters.

Different tools have been developed in recent years. Support for cell tracking has been integrated into commercial applications, such as softWoRx Suite (Applied Precision) and MetaMorph (Molecular Devices), but freely available research tools have also been described such as Particle Tracker  and MtrackJ , two plugins that work within ImageJ [13]. Specific tools have been described to process the paths followed by cells: examples are a trajectory segmentation algorithm, based on supervised support vector classification, or the evaluation of a path according to a brownian model [14,15]. Other methods have been used to describe the population behaviour, often borrowing from techniques used in other fields [16].

Here we present the application of methods for quantitative analysis of the movement of cell populations, which evaluate descriptive statistical parameters and use circular statistics and curve fitting to model directional movement. The methods have been implemented in a software package, which uses an online approach to create an environment where cell tracking, parameter evaluation and statistical analysis is all integrated. This is seen as a web application, MotoCell, which may be conveniently used from the operator desktop, without installation at . The system has been used to study and model the motility of cell populations as well as the behaviour of single individual cells.

## Results and discussion

### Motocell

MotoCell is a web application, designed to track and evaluate the paths followed by cultured cells moving on a surface. Its main goal is to merge a cell tracking module with the ability to statistically evaluate motility of cells or particles. It can directly load both acquisition obtained and stored locally, within the Images database, or external files, organized as a series of frames collected within a folder. Various file formats are accepted (see methods).

**Figure 1:**
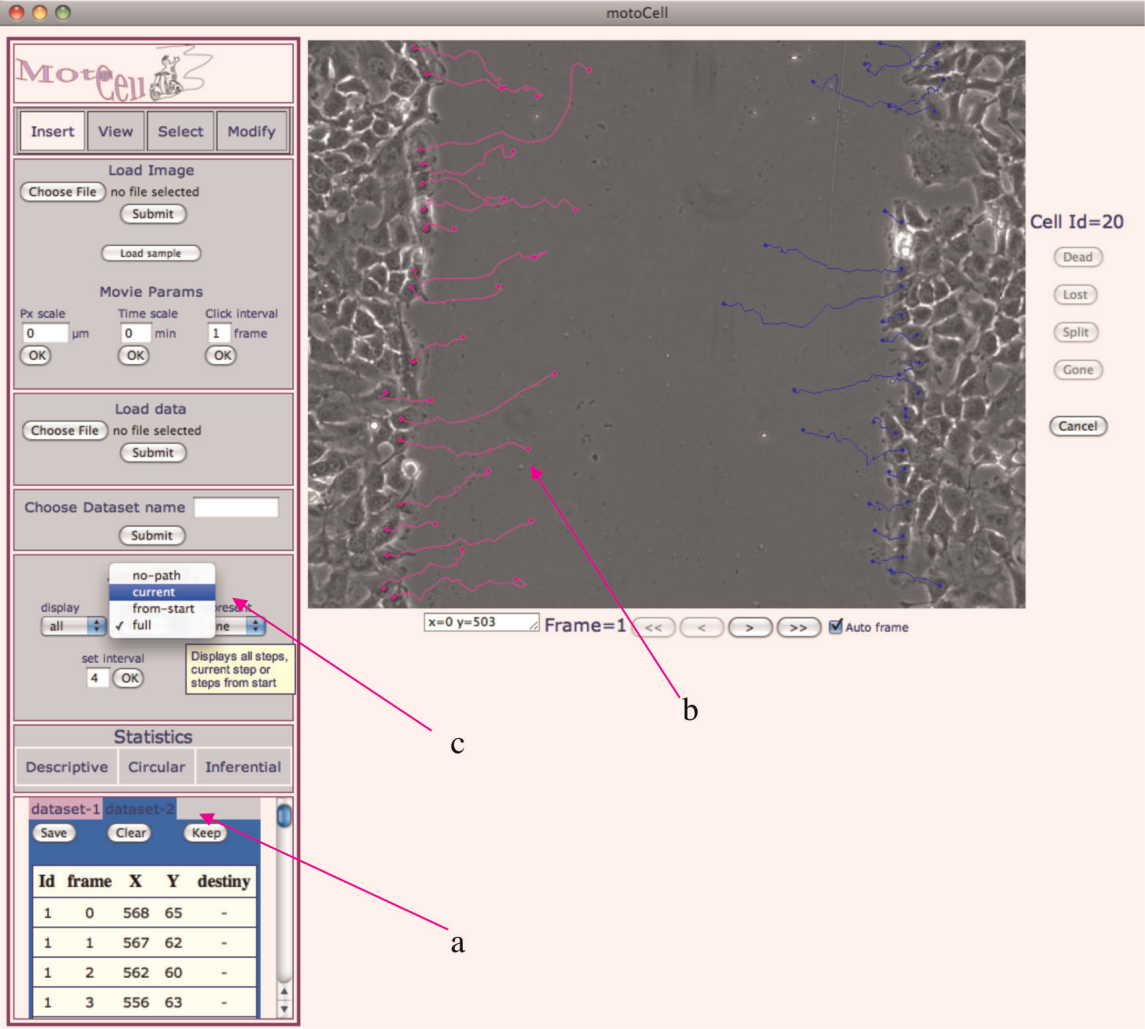
**MotoCell web interface**. The main Motocell interface is used for all operations, including tracking, data handling and evaluation of statistical parameters. in MotoCell assists in cell tracking by recording the users clicks at the various positions in subsequent frames. For each step, coordinates are recorded within the table **(a) **located at the bottom of the Control area and displayed on the image as paths **(b)**. When more datasets are used at the same time, they are alternatively visualized according to the chosen data tab; tab colors are used to match the tables with the corresponding paths on the image. A popup menu **(c) **allows to choose alternative path visualizations: incrementally from the beginning, only at the current time point or as a point sliding along the full path.

The user interface is organized in two main areas, a control panel and an area for visualizing cells and paths and to input cell coordinates (fig. 1). The control panel includes sections containing commands for uploading external acquisitions and change image magnification, to take advantage of the available screen size and better recognize cell compartments or other small structures. The status bar, located immediately below the upload section, is used to select and visualize the 'Insert', 'View', 'Select' and 'Modify' modes. 'View' mode is used to go through the movie, frame by frame or at fixed intervals, whereas the 'Insert' mode is used for input of cell coordinates by clicking on subsequent frames of a movie. Cell coordinates (fig. 1a) for each time steps are stored in text files and used to perform calculations through the web interface. Tables and plots reporting the results of statistical analysis may be downloaded as text or pdf files.

Cell tracking is performed by clicking at the various positions occupied by a moving cell in subsequent frames (fig. 1b); x-y coordinates are recorded and written to a table. The destiny of each cell following its path is also recorded: paths may last for the whole observation time, but may also prematurely end with the death of a moving cell, with a cell split in two as a consequence of a mitotic event, or with the loss of a cell, which moves beyond the limits of the observation field. The coordinates of a path may be modified during or after the tracking phase, in order to correct errors without reclicking the entire data set. 'Select' mode is used to identify the path which needs editing, while the 'Modify' mode is used to assign new coordinates or to change the end of the path. Sometimes researchers are interested in studying subsets of the whole population: the system permits the association of cells to different subsets, which may be separately evaluated (fig. 1b).

**Figure 2:**
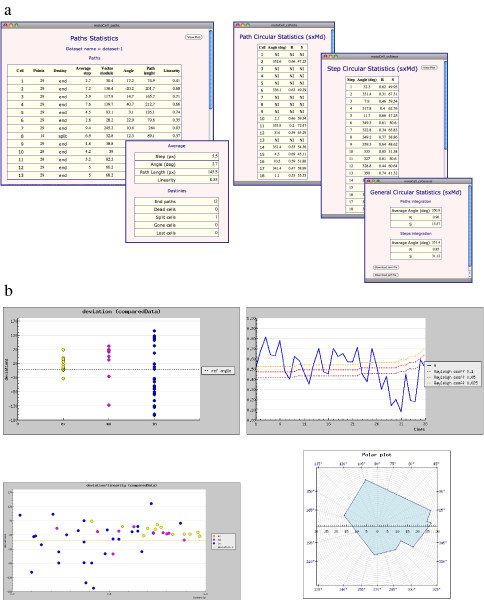
**Output of statistical analysis**. Examples of MotoCell results visualization: a) descriptive and circular statistics parameters, reported for each cell and as average values for the whole population; b) scatter plots, graphs and polar plots, used to show trends in time, path parameters, spatial distribution of directions.

The results of cell tracking, typically stored as a list of cell coordinates in a text file, are evaluated by MotoCell which calculates statistical parameters for each cell path, or the whole population. Sample output windows are reported in fig. 2, where linear and circular statistics parameters are presented as tables (a) or plots (b), designed to quantitatively describe typical experimental situations, as detailed in the following section.

### MotoCell in the study of cell motility

MotoCell is organized around distinct objects. A Movie object takes care of storing and analyzing the behaviour of the whole population. It contains many path objects, which in turn include step objects, corresponding to each elementary movement of a single cell between two contiguous frames. Point and vector objects are used to represent the corresponding physical entities.

**Figure 3:**
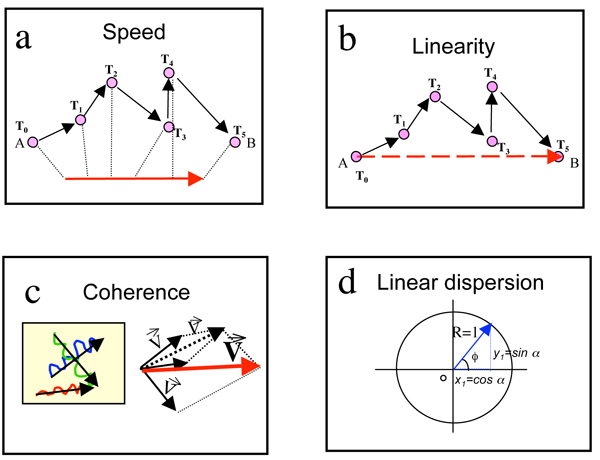
**Statistical parameter evaluation**. a) Speed was calculated as the average of all step lengths; b) linearity as the ratio of net displacement, i.e. the distance between start and end point of a path, to path length; c) coherency of a population is defined as the ratio between length of the resulting vector, obtained by composing the displacement vectors for each cell path, and sum of the single net displacement vector lengths; d) linear dispersion coefficient R is defined as the module of a vector having its origin in the center of a circle with unit radius and direction the average angle *ϕ*.

Within MotoCell, the path length is defined as the sum of all subsequent smaller steps made by a cell, the speed as the average length of all the steps (fig. 3a) performed by a cell within the time interval, and linearity as the ratio of net displacement (i.e. the distance between the starting and end point) to path length. (fig. 3b). For a population, speed is the average of all the steps performed by all the cells, linearity is the harmonic average of linearity independently calculated for each cell.

The average direction of a population is the direction of the resulting vector obtained by composing the displacement vectors for each cell path, while coherency is defined as the ratio between length of the same resulting vector and sum of the single net displacement vector lengths (fig. 3c). Circular statistics analysis is used to evaluate descriptive parameters R and S, by treating the displacements during each step as circularly varying quantities, corresponding to the angle of each displacement vector, and without taking into account its module [16,17]. The average angle describing a path (*ϕ*) is obtained from the arithmetic means of cosine and sine values for each angle (fig. 3d):



where



and



The angle of the obtained average vector has its origin in the center of a circle with unit radius and direction *ϕ*. The module of this vector is named linear dispersion coefficient (R) and may be easily calculated as



R is not dependent on the length of each step, and is descriptive of the distribution of the angles of the step vectors. Its value ranges from 0 to 1, being close to 0 when the angles have a uniform distribution with no directional trend, but gets larger for an asymmetric distribution of angles clustered around a specific direction, reaching 1 for the special case when all the angles are identical (fig. 3d). Angular dispersion (S) is calculated starting from R:



and represents the dispersion of the angles around the average direction.

### Random motility of NIH3T3 fibroblasts

**Figure 4:**
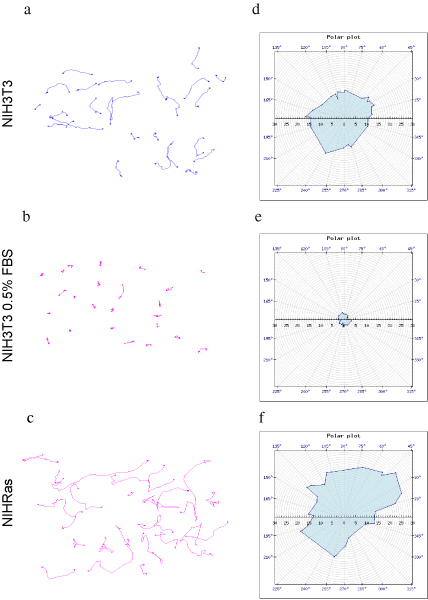
**Random motility analysis**. Paths covered by NIHRas (a) and NIH3T3 in 10% (b) and 0.5% serum (c) moving on the culture surface. (d, e, f) Representation of the paths by using polar coordinates, for the three populations.

In the absence of specific stimuli the movement of cells growing on a culture surface is expected to be randomly oriented in all possible directions and not dependent on time, thus producing a purely random distribution of displacements. Figure 4 shows, on the left, the paths covered by three mouse cell populations maintained in culture plates: NIH3T3 fibroblasts under standard (a) and limiting (b) growth conditions and transformed (NIHRas) by overexpression of Ras oncogene (c) are followed during 10 hours in culture. In order to provide a global representation of the behaviour of each individual cell within a population, polar plots have been generated with MotoCell to graphically represent the distribution of observed net cell displacements. On the right side of figure 4, the directions of the same paths have been represented as a polar graph to graphically visualize their spatial distribution: the randomness of directions can be easily recognized in all cell populations by observing the circular shape of the three areas in the charts. Furthermore, the size of the areas delimited by the polar plots highlights the different ability of the three populations to move away from the starting point. NIH3T3 move much more in 10% serum (d) than under low serum conditions (e); NIHRas (f) show the longest paths.

**Figure 5:**
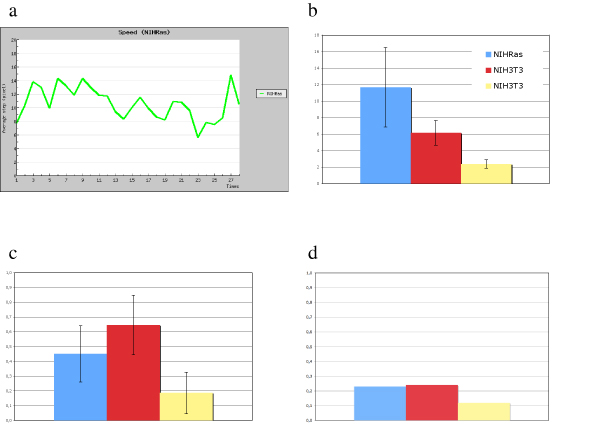
**Evaluation of kinetic parameters**. a) Average step length during the observation time. Plots showing average values for speed (b), expressed as *μ*m/40' step, linearity (c) and coherency (d). NIHRas cells are in blue, NIH3T3 in 10% (red) and 0.5% (yellow) populations.

In order to quantitatively describe movement in better detail, synthetic parameters were calculated within MotoCell for speed, persistence and coherency of movement, by averaging their values at each time point. Although cells obviously change during the period they are kept in culture, time is expected not to be influent as long as a number of conditions are verified: 1) the observation period is kept relatively short, 2) sufficient distance is maintained from critical events, such as culture splitting, cell cycle synchronization, addition of reagents etc., 3) availability of space or nutrients does not become limiting, as for example in cells reaching confluence. These assumptions were verified by observing the behaviour of a NIHRas population, plated at sub-confluent density, starting the observation 12 hours after seeding and prolonging it for 10 hours; the speed values recorded at each time point, tend to remain stable around the average values during the whole observation period as shown in fig. 5a for NIHRas; similar results are obtained for the other lines (not shown). The average speed values, evaluated for NIHRas and NIH3T3 fibroblasts, are reported in figure 5b: NIHRas cells move faster than NIH3T3 under standard culture conditions; speed is further reduced under low serum conditions, as shown for NIH3T3 in the same plot. For all experimental conditions, linearity values are not very high, ranging between 0.2 e 0.6 (c) while coherency is generally low (d), as expected for random movement, where there is no specific reason for preferring one direction rather than another.

### Directional migration of NIHRas fibroblasts

The marked ability of NIHRas cells to migrate on culture surfaces has been studied under different experimental conditions, some of them known to affect the migration of cell populations. For example, when a wound is open within a cell layer, by scratching the surface with a sharp object, the removal of cells from the wounded area acts as a stimulus for the remaining cells to invade and fill the space left empty by the wound. In this type of movement the directional component may be clearly detected as increased coherency and, often, also linearity, as cells moving in a defined direction also tend to maintain the same direction in time (table 1).

**Table 1:** Linearity and coherence of NIH3T3 and NIHRas cell motility

	**NIH3T3**		**NIHRas**	
	Random	Wound	Random	Wound

Linearity	0.44	0.54	0.45	0.62
Coherency	0.24	0.71	0.23	0.8

Values are reported for random motility and wound-healing assays. See details under 'Results'

**Figure 6:**
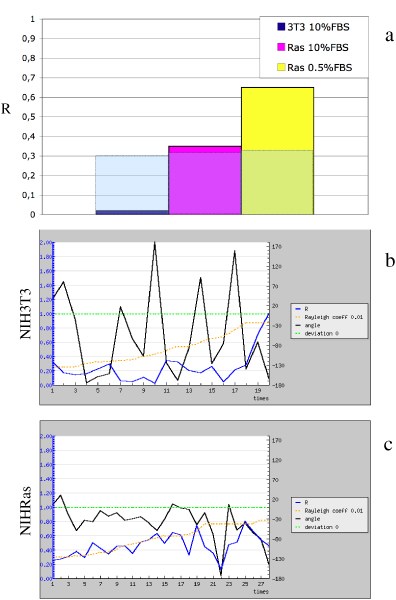
**Evaluation of linear dispersion coefficient (R)**. R coefficients calculated for three cell populations in wound-healing assay (a). Deviation from the expected, i.e. towards the center of the wound, direction and R values are reported for each time point for NIH3T3 (b) and NIHRas (c) populations.

The circular statistics functions within MotoCell have also been used to analyse the directional movement of different cell populations in wound-healing experiments, as reported in fig. 6a. Linear dispersion values show much better directionality for NIHRas (0.365) than NIH3T3 (<0.1) cells; NIHRas score even better under 0.5% serum (0.65), although speed is reduced from 11.5 to 5.2 *μ*/step (data not shown). The significance of parameters evaluated by circular statistics were assessed by using the Rayleigh test [18], which compares the parameters with threshold levels corresponding to values expected for random datasets following the von Mises distribution. Under the used conditions, confidence values better than 0.01 or 0.05, were obtained even for datasets consisting only of a small number of cells. In fig. 6a, the significance levels for P < 0.01 are reported as transparent boxes overlaid onto the histogram.

### NIHRas fibroblast subpopulations in wound healing assays

In order to test the response of the cell layer to a wound, linear dispersion R is plotted in MotoCell as a function of time, together with the average angle, reported as deviation from the expected direction of wound closure. The results obtained for the NIH3T3 population are reported in fig. 6b and show the average direction to be very variable in time, with angles widely ranging between + and -180° of the expected value. Under such condition, R values consistently remain well below the chosen significance threshold, during the whole observation time. The scenery is changed when we consider the behaviour of NIHRas populations (fig. 6c): the deviation angles become close to the expected direction, and R is either above or immediately below the threshold values except at the end of the observation time, when angles start to drift away from the reference, and linear dispersion values go below the threshold, probably reflecting loss of directional movement when the wound is almost closed.

**Figure 7:**
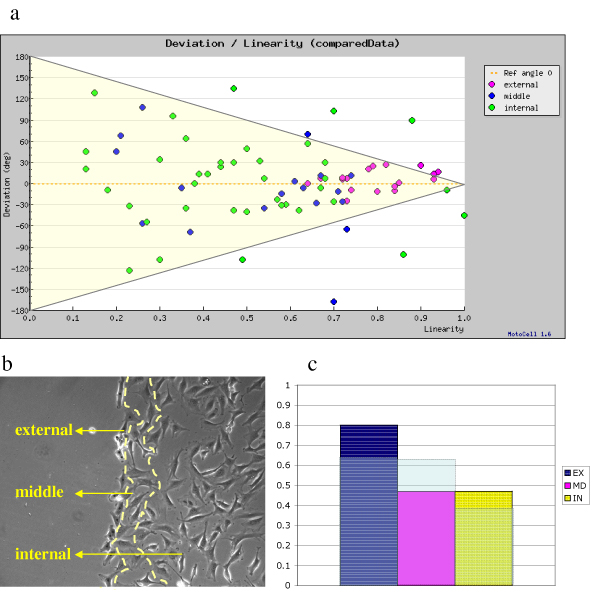
**Relationship between deviation angle and linearity**. a) Deviation from the expected direction, plotted as a function of linearity, in a population subjected to the wound stimulus. b) External, middle and internal sub-populations, identified according to their distance from the wound edge. c) R coefficient for the three sub-populations, compared to the threshold level for P = 0.01.

Linearity of movement in time, usually low or moderate in randomly moving cells, might become higher when cells are stimulated to move towards an attractant or other stimuli. The relation between directionality and linearity was evaluated in the previously described wound-healing assay. The results are shown in fig. 7a where deviation from the reference angle is plotted as a function of linearity. Cells following non linear paths cluster around the left side of the plot and do not show any preferred angle, while those showing high linearity, move according to angles that cluster around the expected direction, i.e. towards the middle of the wound. If different colors are used to distinguish cells located at varying distance from the wound, it is clear that cells showing better linearity and directionality mostly belong to a subpopulation placed at the edge of the wound. Cell with lower linearity are either located in an intermediate position, or far away from the wound. This analysis therefore induces to distinguish different sub-populations, defined as external, middle and internal according to their distance from the wound (fig. 7b). If these sub-population are separately analyzed, the predominat effect of the wound edge on the front population is clearly indicated by the very high R value, well above the threshold for P < 0.01 obtained in circular statistics analysis (fig. 7c).

**Figure 8:**
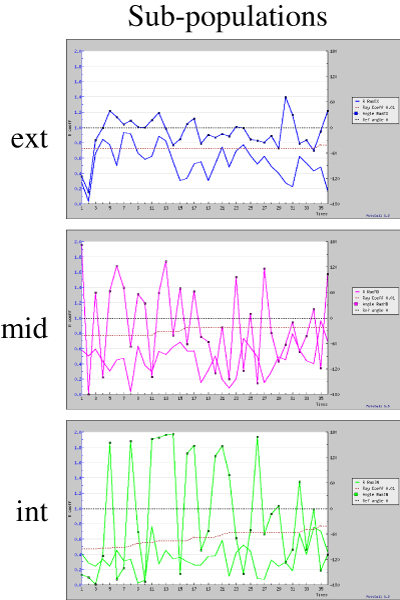
**Time plots for the three subpopulations described in figure 7**. Deviation angle and R coefficient, for the external, middle and internal cell subpopulation. In all plots, expected angle (black) and threshold level for P = 0.01 (red) are reported as a reference.

Time analysis of directional movement of the three separated sup-populations allows to observe that deviations from the expected direction are always small and R values are generally high for the subpopulation close the wound (ext). For the middle subpopulation and much more for the internal one, more scattered values can be observed (fig. 8). This is believed to be a strong indication that the distance from the wound stimulus can modulate cellular movement by acting on directional component of cell migration in a distance dependent fashion.

### Modelling directional movement with von Mises distribution

**Figure 9:**
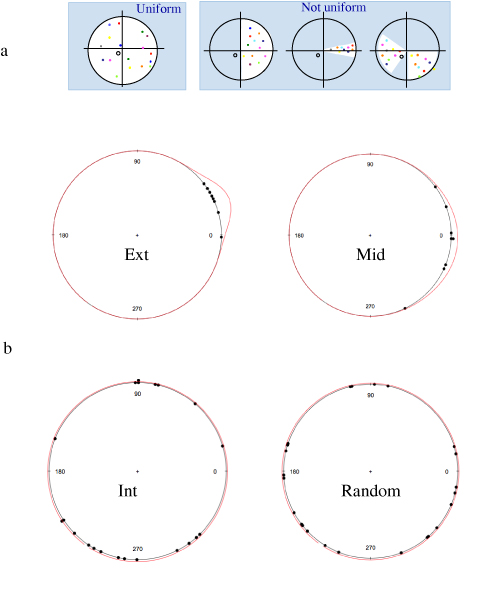
**Circular plot of path directions**. a) Examples of uniform and non-uniform distributions. b) Curves describing the best fitting von Mises distributions, overlaid onto the experimental points for external, middle, internal, and random populations.



Circular statistics detects the not uniform distribution of a given dataset, but is not able to discriminate between different non-uniform distribution models, as shown in fig. 9a. The von Mises distribution is commonly used as a model for many circular data problems. It fits well to points tightly concentrated around a mean direction. It is defined by:

where 0 ≤ *ϑ *< 2*π*, *κ *≥ 0, 0 ≤ *μ *< 2*π *and  is the zeroth Bessel function, *μ *indicates the mean direction and k the concentration parameter, in some way recalling the gaussian distribution [19].

In order to attempt to model the behaviour of a cell population according to a von Mises distribution, the net displacements of each cell following a path have been used to compute the maximum likelihood estimates for the parameters of a von Mises distribution. Overlays of the theoretical curve with the experimental data are reported in fig. 9b, while the calculated parameters are in table 2.

**Table 2:** Fit to von Mises distribution

	*μ*(degree)	*κ*	K	Cr. value vM	Test Circ.	Cr. value Circ.
external	21.62 +/- 4.385	19.48 +/- 9.056	**0.073**	0.164	0.625	0.267
middle	-6.49 +/- 10.12	4.542 +/- 2.084	**0.037**	0.158	0.395	0.267
internal	-99.43 +/- 43.23	0.4133 +/- 0.319	**0.060**	0.09	**0.113**	0.267
random	-122.4 +/- 56.13	0.2963 +/- 0.293	**0.036**	0.09	**0.058**	0.267

First and second columns report mean direction *μ *and concentration k for best fitting von Mises distributions, respectively for external, middle, internal and random populations. In columns 3 to 6, results for von Mises (3) and uniform (5) tests are reported, next to their critical values (4 and 6) for the populations described in fig. 9

In order to evaluate the fit of the calculated model to the experimental data, the Watson test was used to test for both a von Mises and a uniform distribution [20]. The results are reported in table 2, where for all tested datasets the hypothesis of von Mises distribution may be accepted while the uniform hypothesis should be rejected for the external and middle population. In all cases a significance level of 0.01 was chosen. It should be noted that fitting to a von Mises is compatible, but not indicative of unidirectional movement, because a very wide and flat distribution is still acceptable as a von Mises. Of course such a distribution is easily recognized, as it would also fit a circular model and produce lower linear dispersion values in the circular statistics test. With this approach, a bimodal distribution, is also easily recognized as it may result acceptable as a circular, but not as a von Mises distribution model (not shown).

## Conclusion

Cell migration is involved in important processes in embryo development and adult life and is mediated by a very complex machinery, which includes a large number of membrane bound, soluble and nuclear factors. The web application presented here, MotoCell, may be used to describe the behaviour of both single cells and whole cell populations by separately analyzing and quantitatively evaluating parameters, descriptive of speed and directionality of cell movement. MotoCell integrates all the relevant tasks within a unique environment, where cell tracking, plot generation and statistical evaluation may be quickly and easily performed. The software, originally developed as a collection of scripts for single user PCs, in its present web form, offers important advantages, such as tight integration with a shared image database, and no need for data transfer between hosts before analysis. Although based on a standard web interface, by exploiting the web approach in a very effective way, the program results very fast and interactive and is immediately available to a large number of users.

The described case study allowed to analyze the directional movement of NIHRas transformed cells as a function of time and in relation to stimuli. Statistical parameters describing consistency of directional movement in time (linearity) and across the cell population (coherency) were evaluated for this cell line, together with circular statistics parameters as linear dispersion coefficient (R) of the cell paths and angular dispersion (S) values around the average angles. The results clearly show the Ras transformation increases both speed and directionality of cell movement.

The ability to evaluate the behaviour of single cells allows to draw the attention on specific correlations, such as persistence of movement and deviation from the expected direction as shown in figures 6, 7, 8. By using the Rayleigh test to assess the significance of circular statistics parameters, it was possible to recognize that confidence limits better than 0.01 may be achieved in tests involving even a limited number of cells. Fitting the observed data to the von Mises model, as well as to the circular model, allowed to decide whether an observed non-uniform directional movement, determined by circular statistics and directed towards a given direction, is correctly assigned to a unidirectional model.

The application of these methods to the study of fibroblast movement supports a relationship between cell path linearity and population coherency in many experimental situations and allowed to detect the existence of defined subpopulations, located at increasing distance from the wound edge and characterized by different motility features.

## Methods

Most analysis are performed within a web application, MotoCell, which is used to track moving cells by interactively clicking at the various positions occupied by them in subsequent frames; x-y coordinates are recorded and written to a table. The destiny of each cell following its path is also recorded: paths may end with the death of the moving cell, with a mitotic event, where the cell is split in two or by loss of the cell, which goes out of the observation field. The paths are shown superimposed upon the image and grow while the movie progresses towards the final frame. The coordinates of the recorded paths are saved to a text file, which may be stored for further analysis. Editing of the coordinates is possible both during and after the tracking phase; to correct errors without reclicking the entire set of data.

MotoCell calculates descriptive parameters such as average speed and linearity, along with circular statistics analysis of linear and angular dispersion. Statistical analysis is performed by using the saved coordinates list, either just acquired or stored in previous sessions. MotoCell is accessible online at the address ; users datasets may be uploaded from the remote client PC. Statistical analysis is performed by using the saved coordinate lists.

Analysis of cell behaviour consists of the evaluation of the parameters of descriptive statistics such as average speed, linearity, mean angle, vector length, calculated for the whole population examined as well as for each cell and for each step of the migration; in this way the behaviour of whole cell population may be assessed as a whole or as a sum of individual entities. In addition to population statistics, the analysis of single cells allows to group the cells into subpopulation, or even to evaluate the behaviour of each cell with respect to a variable reference, such as the direction of a wound or the position of the closest cell.

Circular statistics analysis is used to study the directional movement of objects and produces linear dispersion coefficient (R) and angular dispersion (S) values, together with average angles. The data are examined in relation to the Rayleigh coefficient for evaluating the significance of data derived from small cell populations.

## Competing interests

The authors declare that they have no competing interests.

## Authors' contributions

CC, LS and GP thought and designed the MotoCell application and its interface and gave major contribution to the writing; CC and FF developed most of the code; LS and MCF carried out the wet-lab experiments, while GP provided oversight of the work.
